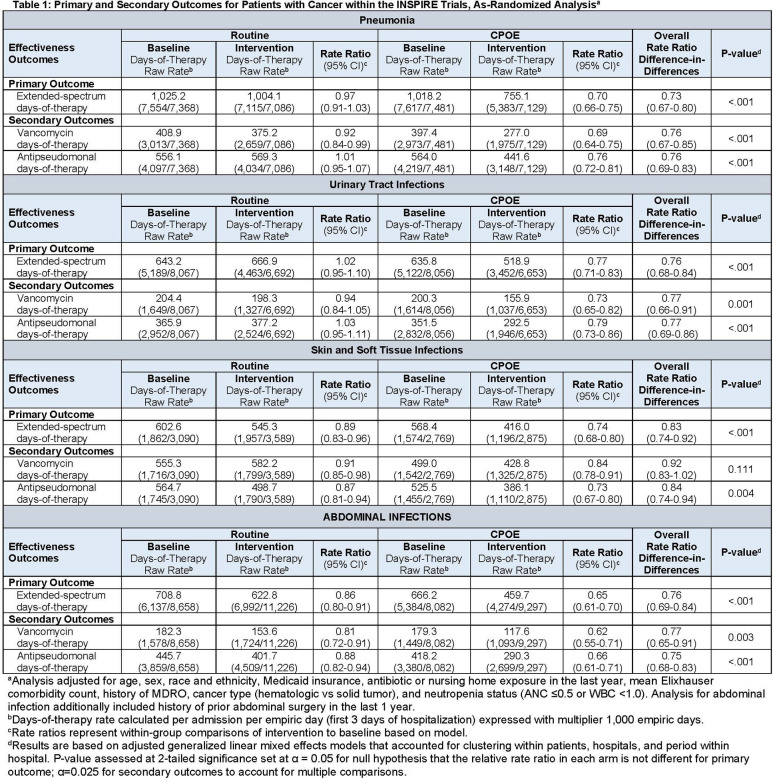# 23 Partnering for Prevention: A Public Health & Academic Collaboration to Address Measles Outbreaks in Kentucky

**DOI:** 10.1017/ash.2026.10431

**Published:** 2026-06-23

**Authors:** Shruti Gohil, Taliser Avery, Ken Kleinman, Ed Septimus, Amarah Mauricio, Kenneth Sands, Neha Varma, Selsebil Sljivo, Kaleb Roemer, William Cooper, Russell Poland, Robert Weinstein, Samir Fakhry, Jefrey Guy, Julia Moody, Micaela Coady, Kimberly Smith-Sells, Mary Hayden, David Kubiak, Chenette Burks, Richard Platt, Susan Huang, Meghan Baker

**Affiliations:** 1 University of California, Irvine; 2 University of Massachusetts, Amherst; 3 Harvard Medical School and Harvard Pilgrim Health Care Institute; 4 Division of Infectious Diseases, University of California, Irvine School of Medicine, Irvine, California; 5 Hospital Corporation of America; 6 Department of Population Medicine, Harvard Pilgrim Health Care Institute, Boston, Massachusetts; 7 HCA Healthcare; 8 Rush University Medical Center; 9 Frist College of Medicine, Belmont University; 10 Harvard Pilgrim Health Care Institute; 11 Brigham and Women’s Hospital; 12 HMS/HPHC; 13 University of California Irvine School of Medicine; 14 MGB, DFCI

## Abstract

**Background:** Empiric extended-spectrum antibiotics are routinely given to patients with cancer despite low risk of infection with multidrug-resistant organisms (MDROs). This secondary analysis of the four INSPIRE (INtelligent Stewardship Prompts to Improve Real-time Empiric Antibiotic Selection) trials evaluated how computerized physician order entry (CPOE) prompts providing patient- and pathogen-specific MDRO risk estimates affected empiric extended-spectrum antibiotic use in patients with cancer. **Methods:** We identified non-critically ill hospitalized adults (> **Results:** Including all trials, 36,861 (18,272 baseline; 18,589 intervention) patients had cancer. Mean age was 69.0 (13.6); 48.0% (17,675) were male. Extended-spectrum antibiotic days-of-therapy decreased by 27% (95% CI:20-34%, PPPP Conclusions and Relevance: An antibiotic stewardship bundle that included CPOE prompts recommending standard-spectrum antibiotics for patients at low risk for antimicrobial-resistant infections reduced extended-spectrum antibiotic use in non-critically ill patients with cancer who were hospitalized with community-acquired pneumonia, UTI, abdominal infection, or SST, without observed differences in safety outcomes.

**Figure:**